# Testing the Role of Natural and Sexual Selection on Testes Size Asymmetry in Anurans

**DOI:** 10.3390/biology12020151

**Published:** 2023-01-18

**Authors:** Shengnan Chen, Ying Jiang, Long Jin, Wenbo Liao

**Affiliations:** 1 Key Laboratory of Southwest China Wildlife Resources Conservation (Ministry of Education), China West Normal University, Nanchong 637009, China; 2 Key Laboratory of Artificial Propagation and Utilization in Anurans of Nanchong City, China West Normal University, Nanchong 637009, China

**Keywords:** anurans, body size, environmental stress, natural selection, sexual selection, testes size asymmetry

## Abstract

**Simple Summary:**

Testis asymmetry is particularly common in animals and it has been explained by two main hypotheses: the packaging hypothesis and compensation hypothesis. We studied the variations in testes size asymmetry among 116 anuran species associated with natural and sexual selection to test the two hypotheses. We found that the positive correlation between testes size asymmetry and livers mass followed the prediction of the packaging hypothesis, while the postcopulatory sperm competition (e.g., residual testes size) and the degree of testes asymmetry was positively related, supporting the positive role of sexual selection on testes size asymmetry. However, we did not find any effect of developmental stress on variation in testes size asymmetry among anurans, which was inconsistent with the compensation hypothesis.

**Abstract:**

Directional asymmetry in testes size is commonly documented in vertebrates. The degree of testes size asymmetry has been confirmed to be associated with natural and sexual selection. However, the role of natural and sexual selection driving variations in testes size asymmetry among species of anurans are largely unknown. Here, we studied the patterns of variations in testes size asymmetry and the factors shaping its variations among 116 anuran species. The results indicated that the left size-biased testes in 110 species (94.83% of 116 species) is more common than the right size-biased testes in six species. For all studied species, the degree of testes size asymmetry was positively associated with relative livers and body fat mass, following the prediction of the packaging hypothesis. We also found that the postcopulatory sperm competition (e.g., residual testes size) was positively associated with the degree of testes asymmetry. However, environmental stress (e.g., high latitude, precipitation seasonality and temperature seasonality) did not promote more symmetrical testes for all species. Our findings suggest that both natural selection for larger livers in body space and sexual selection for rapid increase in testis mass for most species during the breeding season can play key roles in driving in testes size asymmetry across anuran species.

## 1. Introduction

Directional asymmetry in gonad size and/or shape is first documented in birds at least 220 years ago [1], and it is also widespread in the animal kingdom [2,3,4,5,6,7,8,9,10,11,12,13,14,15,16,17,18,19,20]. Testis asymmetry is particularly common within or among species of frogs and birds, and thus leading to directional biases in size and/or shape [4,21]. Two main hypotheses have been suggested to explain the testes size and/or shape asymmetry within and among species. The packaging hypothesis (PH) predicts that testes asymmetry reflects space constraints of the body cavity, in particular liver asymmetry in males [22]. Thus, asymmetrical livers may constrain the available space for the testis growth. As a result, the degree of testes asymmetry among species reflects differences in selection pressures on the arrangement of the body cavity. The compensation hypothesis (CH) states that one testis should grow more than the other testis where it would be expected to compensate for a reduced function within the species [23]. This functional explanation assumes that intraspecific variation in the degree of testes asymmetry reflects the situation where the one testis size increases due to the malfunctioning of the other testis [2].

A common situation of testes size asymmetry is that the left testis often develops to be larger than the right testis [23]. Such asymmetry can be considered as a result of natural and sexual selection (reviews in [24]). Specifically, the constraints of body space for a larger right lobe of the livers and natural selection on gonads in females, where the right gonad is almost always degenerate in favor of a high degree of left size-biased testis asymmetry [18]. By contrast, sperm competition is an important evolutionary force driving variations in primary sexual traits such as testes size and histology [20,25,26,27,28]. Consequently, postcopulatory sexual selection results in more symmetrical testes in order to maximize sperm production. Indeed, there is evidence that the level of sperm competition promotes more symmetrical testes across 67 bird families because sperm competition selecting for the increased mass of sperm-producing tissues may be more efficient when sperm production occurs in two testes simultaneously [24]. Alternatively, because of the rapid increase in testis size in the breeding season, the physiological efficiency is expected to favor the enlargement of one testis, and the increases in testes size will lead to a higher degree of testes size asymmetry [24].

Interestingly, intra- and inter-specific studies on the evolutionary causes of testes size asymmetry associated with environmental stress (e.g., latitude and diet) have been investigated recently in frogs and birds [4,24]. For instance, latitude is not correlated with intraspecific variation in testes size asymmetry among 22 *Rana temporaria* populations along a 1,600-km latitudinal transect, although environmental conditions and presumably environmental stress display greatly differences [4]. Likewise, seeds in the diet are not associated with interspecific variation in testes size asymmetry among species of birds [24]. However, the variations of testes size asymmetry underlying natural and sexual selection and environmental stress among species of anurans is yet unknown.

Our aims in the present study were to investigate interspecific variations in testes size asymmetry and the roles of natural and sexual selection and environmental stress on size asymmetry among anuran species. First, we explored the prediction of the packaging hypothesis that natural selection for increased livers should favor a higher degree of testes size asymmetry due to the constrained availability of space in the body cavity [22]. Second, because of sperm competition more efficiently selecting for an increase in testes mass when sperm production occurs in two testes simultaneously [24], we expected a negative relationship between the degree of testes size asymmetry and the level of sperm competition (e.g., residual testes size). Finally, as individuals with symmetrical testes are assumed to be associated with increasing developmental stress [2], we expected the prediction of the compensation hypothesis that species living with higher environmental stress (e.g., latitude, precipitation seasonality and temperature seasonality) should display lower level of testes size asymmetry.

## 2. Materials and Methods

### 2.1. Data Collection

**Figure 1:**
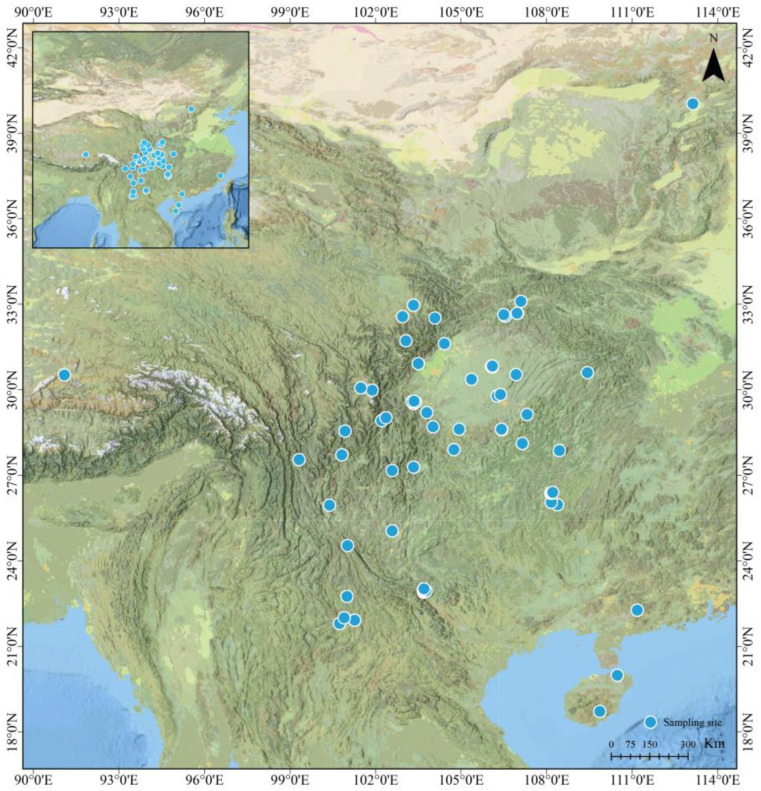
Distribution map of sampling site.

A total of 116 species of anurans (469 individuals (N = 4−6; mean: 4.04 per species)) from eight family were collected in breeding seasons between 2010 and 2022 in Southern and Western China (Figure 1). We sampled each species in a single site by the sampling-lined method, and recorded their locations including altitude and latitude. All individuals were captured along a sampling line at night using a 12-V flashlight. For all individuals we confirmed adults and sex based on their secondary sexual characteristics (e.g., gonads in females and nuptial pads in males [29]). Body size (snout-vent length: SVL) of each individual was measured to the nearest 0.01 mm with a caliper. Subsequently, we used single-pithing to euthanize each individual and then preserved them in 4 % buffered formalin. After two months of preservation, the left and right testis and livers of each individual for all 116 species were dissected, and we weighed them to the nearest 0.1 mg using an electronic balance. For collection of adult sex ratio (ASR: defined as the number of males to the number of fertilizable females) per species of 116 species of frogs, we searched a line transect, 5 m wide and on average 1.3 km (range: 0.4–4.6 km) long using a 12-V flashlight and recorded the number of male and female frogs in successful three days [30,31]. We calculated the ASR per species based on the male and female number of sampling lines (Table S1). All procedures were approved by the management office of China West Normal University Ethical Committee for Animal Experiments.

### 2.2. Associated Variables

Polygamous species have more larger scope for mating competition than monogamous species, and thus sperm competition is stronger in polygamous species than in monogamous species [27,32,33,34,35,36]. We categorized mating system as either polyandry that a female was clasped by multiple males and sperms from males simultaneously compete for the eggs or monandry where a male mates with one female over the course of a breeding period according to the sources [27]. Latitude affects significantly variations in testes mass among populations in frogs [4,35], we therefore extracted information on latitude that is typically associated with testes mass (Table S1). In addition to latitude, climate variables have been hypothesized to affect morphometric symmetry within-species evolutionary processes [2]. Hence, testes size asymmetry was expected to be affected by environmental harshness. Here, we used temperature seasonality (standard deviation ×100) and precipitation seasonality (coefficient of variation) as environmental harshness. We obtained precipitation seasonality and temperature seasonality from WorldClim v2 [37], and extracted environmental predictors for each studied location using ArcGIS 10.8 [38]. All individuals measured in each species were processed at same moments.

### 2.3. Phylogeny Reconstruction

**Figure 2:**
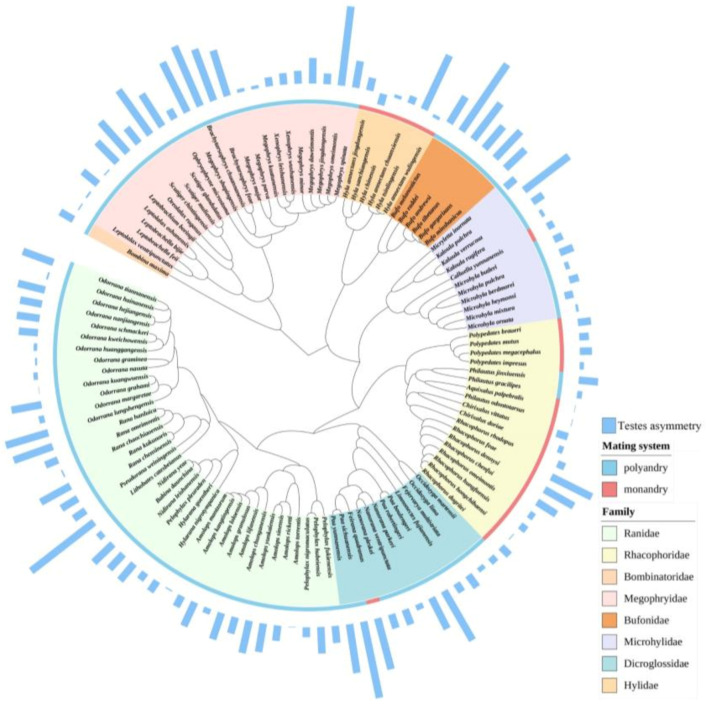
Phylogenetic tree of the 116 anuran species based on the three nuclear genes (RAG1, RHOD and TYR) and the six mitochondrial genes (CYTB, 12S, 16S, COI, ND2 and ND4) using BEAUTi and BEAST v.1.8.3. Blue histogram indicates the degree of testes size asymmetry.

We reconstructed the phylogeny based on the DNA sequences of three nuclear genes (RAG1, TYR and RHOD) and six mitochondrial ribosome genes (COI, CYTB, 16S, 12S, ND2 and ND4) downloaded from NCBI GenBank for all species of anurans because the nine genes were unity for all species (Table S2). We used multi sequence alignment (MUSCLE) in MEGA v.11 [39,40] to align the sequences, and then determined the best nucleotide substitution model by using the function modelTest in the R package phangorn v.2.8.1 [41] based on the Akaike information criterion. We used GTR+Γ+I as the best substitution model for all genes. The GTR+Γ+I was regarded as best nucleotide substitution model for all genes except RHOD, for which the stronger support is HKY+Γ+I. We used BEAUTi and BEAST v.1.8.3 ([42]; also see details in [43]) to construct the anuran phylogeny, and the Markov Chain Monte Carlo (MCMC) simulation was run for 100 million generations where we used the BEAST implementation in the CIPRES Science Gateway (http://www.phylo.org, accessed on 15 September 2022) every 10,000th tree to sample. Finally, maximum clade credibility trees were generated with mean node heights and a 10% burn-in for 116 species using TreeAnnotator v.1.8.3 ([42]; Figure 2).

### 2.4. Statistical Analysis

All analyses were conducted on log_10_-transformed data in R statistical version 4.2.0 [43]. We first taken traits all estimated at species-level mean and then estimated the testes size asymmetry. We defined testes size asymmetry as TA = log_10_ (left testis)-log_10_ (right testis) because it is positive for a left-biased size and negative for a right-biased size. The residual testes size was estimated as residuals from the regression of log_10_-transformed SVL and log_10_-transformed combined testes mass. Combined testes mass was the sum of the mass of the right and the left testis.

We constructed phylogenetically general least squares models (PGLS) using the pgls function in the R caper ver. 1.0.1 [44] to control for the non-independence of data. In each model, we used the maximum-likelihood estimate of phylogenetic dependence the *λ* -statistic with its 95% confidence limits (CL) to confirm phylogenetic correlation. When *λ* = 0 none of the variation can be accounted for by phylogenetic relationships, and when *λ* = 1 all of the variation is explained by phylogeny alone [45,46,47]. The degree of deviation of *λ* from these null models was determine based on likelihood ratio tests, and all PGLS models were tested with estimated *λ* values against models with *λ* fixed at either 0 or 1 [48]. We reported the P-values of these tests as superscripts following the *λ* values. We calculated effect sizes and their non-central 95% CLs as partial r for continuous following Nakagawa and Cuthill [49]. For all PGLS models we chose SVL as a covariate because it the commonly used measure of body size in anurans and is independent of seasonal fluctuations in tissues such as body fat, testes, or limb muscles.

Because the sample size for each species ranges from four to six individuals, and it is likely to exist error in the measurements of left and right testis mass. However, as many as 113 species have pertaining to four individuals, and 3 species have data from 5–6 individuals, the measurement error or intraspecific variation should not play a large role in the estimate of testes asymmetry. We used Brownian Motion to test for best fits our data before running PGLS. We first applied a separate PGLS model to test interspecific associations between testes size asymmetry and combined testes mass when controlling for effect of SVL. We then built multi-predictor models to test interspecific association between testes size asymmetry and livers, lung and body fat mass across species when controlling for effect of combined testes mass and SVL. Third, we ran multi-predictor models to test the relationships between testes size asymmetry and environmental harshness (e.g., latitude, temperature seasonality and precipitation seasonality) after correcting for SVL and combined testes mass. Finally, we ran one additional separate analysis to test whether ASR and/or mating system affect variation in testes size asymmetry when controlling for SVL effect. We standardized all numeric variables before analyses to make parameter estimates comparable, and model assumptions were also checked and met.

## 3. Results

**Figure 3:**
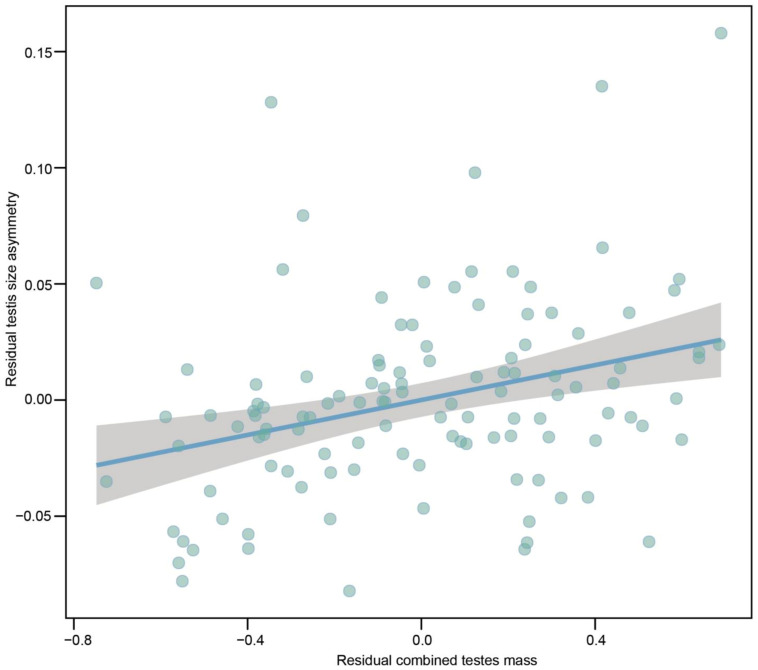
Partial regression plot of residual testes size asymmetry on residual combined testes mass when controlling for phylogeny and SVL across 116 species of anurans. SVL and combined testes mass were log_10_-transformed. Species with higher residual combined testes mass are expected to encounter more intense sperm competition.

With respect to testes size, a left bias was more common than a right bias for 110 species (94.83% of 116 species), while the right bias in 5.17% (n = 6) of the 116 species. The average size of the left testis was a greater value than the right one among 116 species (*t*_113_ = 2.064, *P* = 0.041; *λ* = 0.652^1.00,<0.01^), displaying a left bias testes asymmetry. We examined the relationship between indices of sexual selection (e.g., relative testes size, ASR and mating system) and testis size asymmetry. PGLS models revealed that the degree of residual testes size asymmetry was positively correlated with interspecific variations in residual combined testes mass when controlling for phylogeny and SVL (Figure 3; Table 1). Thus, testes are more size-asymmetrical in species with more intense sperm competition. We did not find a positive correlation between residual testes size asymmetry and SVL. However, none of indices of sexual selection (e.g., ASR and mating system) can explain variations in degree of residual testes asymmetry among species when controlling for phylogeny and SVL (Table 1; Figures S1 and S2). The degree of residual testes size asymmetry was positively correlated with SVL for the two models (Table 1).

**Figure 4:**
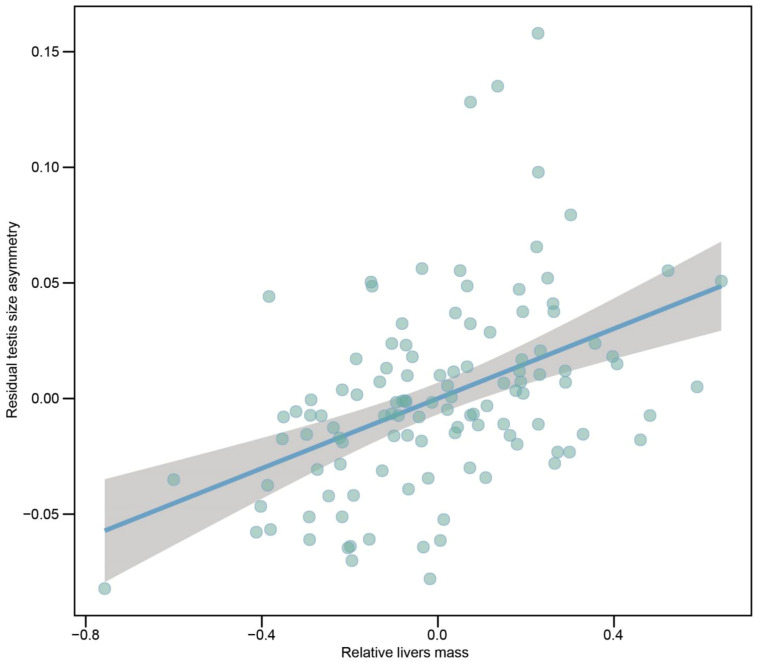
Partial regression plot of residual testes size asymmetry on residual livers mass when controlling for effects of combined testes mass, SVL and phylogeny across 116 species of anurans. SVL, combined testes mass and livers mass were log_10_-transformed.

We examined the relationship between index of natural selection and testis size asymmetry using PGLS models. For all species, the degree of residual testes size asymmetry was positively correlated with residual livers mass when controlling for effects of SVL, combined testes mass and phylogeny (Figure 4). Across all species, a relatively larger left testis was associated with a relatively larger liver mass. However, we did not find a positive relationship between degree of residual testes size asymmetry and SVL (Table 1). We also found a positive relationship between residual testes size asymmetry and residual body fat mass and a positive trend between residual testes size asymmetry and residual lung mass (Table 1).

Testes size asymmetry was not correlated with environmental harshness (latitude, precipitation seasonality and temperature seasonality), controlling for SVL, combined testes mass and phylogeny (Table 1; Figures S3 and S4). Hence, the interspecific variations in residual testis asymmetry cannot be explained by latitude and environmental harshness. Moreover, there were not positive correlations between residual testes size asymmetry and SVL for the two models (Table 1).

## 4. Discussion

Our study indicates the assumption that a larger left testis is the more common trait in anuran species. The occurrence of a right size-bias in 5.17% of species indicates that a left size-biased testis can be possibly the result of a related response to selection on gonad asymmetry in females. Consistent with the prediction of the PH, we find a positive correlation between residual testes size asymmetry and residual livers and fat body mass among 116 species of anurans. We also find a positive relationship between residual testes size asymmetry and residual testes size, suggesting that because of rapid increase in testis size, physiological efficiency in the breeding season favor enlargement of one testis rather than two testes. However, the degree of testes size asymmetry cannot be explained by the indices of precopulatory sexual selection (e.g., mating system and ASR) and environmental stress (e.g., latitude, precipitation seasonality and temperature seasonality).

Testes size asymmetry is particularly common and often pronounced in birds (e.g., [23,24,50]) as well as anurans [4,8,13]. For birds, left bias in testis size is the common pattern observed across species (75% of cases), which occurs in three times as many species as those with a larger right testis [24]. Moreover, previous studies have indicated that most species of anurans display the intraspecific patterns of left size-biased testes asymmetry [8,10,13]. Indeed, the occurrence of the left size-biased testes in 94.83 % of species in this study suggested that strong selection pressure for larger left testis size can be achieved only by growing the less space-constrained right testis. We found testis size variation across species which can be explained for two reasons: (1) polyandry is an obvious selection pressure favoring the evolution of bigger testes and packing arguments—where can we fit in more testicular tissue; (2) egg deposition systems may also affect effective sperm transfer, for instance, foam nesters (many Rhachophorid species) may have very effective sperm transfer into the foam nest—meaning less pressure to increase ejaculate volumes/testis size versus aquatic egg deposition (e.g., Bufonids) where sperm dispersal may occur into the water around mating pairs.

A comparative study for the role of the PH on testis asymmetry suggests that the degree of testes symmetry is correlated with space, which is constrained on the left side of the body cavity where a large gizzard in granivores occur [24]. Specifically, testes asymmetry responds to space constraints for the livers and gizzard in the male body cavity where the positioned asymmetrical organs limit the space available for the growth of each testis [22]. Following the prediction of the PH, we found that the correlation between degree of testes size asymmetry and livers, and body fat mass (i.e., the proxy of space available for the growth of testis) across species was positive, suggesting that the left size-biased testes symmetry resulted from the fact that the right size-biased livers and body fat restricted the space available for the growth of right testis in anurans. Especially, body fat is an essential feature of anuran viscera in burrowing species with extensive periods of hibernation/aestivation. Although maximum lung volume may be a bigger constraint on the size of internal organs in frogs given the essential role of lungs in call production in males, we did not find a significant relationship between testis asymmetry and lung mass. Furthermore, selection on one side of female gonads in the constrained space favors a higher degree of testes asymmetry [25]. However, we cannot provide evidence for female gonad symmetry in anurans. Clearly, more work is needed to solve the view of whether female gonad symmetry reflects variations in testes size asymmetry in frogs.

The compensation hypothesis was developed to explain within-species variation in directional testis size asymmetry [2]. It can be explained by two sides: (i) the idea that higher quality individuals (using a secondary sexual trait as proxy) have more asymmetric testes; and (ii) the idea that extreme asymmetric individuals can have comparable total testes mass (proxy for sperm producing tissues) to the average in the population. Although body condition is listed as male quality in birds that could affect testes asymmetry because of poor function in males with poor condition [7], the lack of association between degree of testes asymmetry and male quality in frogs fails to support the prediction of the CH [8,10,13]. Estimates of body condition as a species-specific male quality cannot be compared across species in that way. Hence, the present study cannot attempt to test the CH by analyzing association between testes asymmetry and body condition among anuran species using a comparative approach. Indeed, we only found positive associations between the degree of testes asymmetry and SVL for two models, suggesting that SVL cannot be interpreted as a proxy measure of body type/shape across species of anurans, although SVL often is a trait that could have relevant biological meaning in the context of internal organ arrangement [51].

Interestingly, to maximize sperm production, the postcopulatory sexual selection leads to more symmetrical testes [14]. For instance, postcopulatory sperm competition affects slightly the degree of testis size asymmetry in birds as about 6% of the observed variations in testes size asymmetry can be explained by relative testis mass [7]. Hence, postcopulatory sexual selection might result in variations in testis size asymmetry due to its effect on the combined testes size and internal tissue structure [28]. Previous studies have indicated that postcopulatory sperm competition promotes an increase in relative testes size [27,52,53] and affects the internal tissue structure of testes in frogs [54]. Postcopulatory sexual selection, hence, was expected to affect the degree of testis size asymmetry in anurans. In the study, we found a positive relationship between testis size asymmetry and combined testes mass, suggesting that sperm competition can be regarded as a selective force, making testes more asymmetrical in size because the developmental advantage to enlarging one testis is linked to less energetic and physiological costs for the increases in total testes mass. However, the non-significant correlations between testis size asymmetry and mating system and/or ASR suggested that precopulatory sexual selection cannot drive variations in testis size asymmetry in anurans.

There are evidences that environmental harshness has been suggested to as the important factors affecting morphology asymmetry in taxa [55,56,57,58,59]. A similar interpretation can be applied to directional morphometric symmetry where individuals with larger degree of asymmetry can deal with current environmental conditions, and thus can be associated with a lower level of climate variables [2]. However, there was no correlation between latitude and the level of directional asymmetry in testis size among populations in a frog [4]. Here, we found that environmental harshness (e.g., latitude precipitation seasonality and temperature seasonality) did not affect the level of testes size asymmetry among anuran species. Hence, our findings were not consistent with the compensation hypothesis that developmental stress can shape anuran testes asymmetry in testes size.

## 5. Conclusions

In summary, we detect directional testis size asymmetry across 116 anuran species and offer hypotheses that could explain the observed patterns. We first reveal that the degree of testes size asymmetry is dependent on livers and body fat mass among species of anurans, which is consistent with the prediction of the PH. Furthermore, postcopulatory sexual selection can explain the patterns of testes size asymmetry due to physiological efficiency. However, neither precopulatory (i.e., mating system and ASR) nor developmental stress can explain patterns of testes size asymmetry. The study needs more species and samplings on the effect of impacted factors on testes size asymmetry in frogs.

## Figures and Tables

**Table 1:** Models predict testis size asymmetry when controlling for phylogeny effect using the PGLS function in the R package caper [44]. Lambda (*λ*) estimates the phylogenetic signal using maximum likelihood. Effect sizes were calculated following Nakagawa and Cuthill [49]. Effect sizes and their non-central 95% CLs were calculated as partial *r* for continuous predictors. For each model estimates of the coefficients for each effect are listed, and significance levels tested with the *t*-statistic.

Association	Number of Species	*λ* (95% CL)	Adjusted R^2^	Effect Size (95% CL)	Estimate (+SE)	*t* (*P*)
1. Size asymmetry	116	0.127 (0.006, 0403)	0.452			
Combined testes mass				r = 0.322 (0.148, 0.468)	0.038 (+0.011)	3.614 (<0.001)
SVL				r = 0.177 (−0.007, 0.343)	0.079 (+0.041)	1.910 (0.059)
2. Size asymmetry	116	0.114 (<0.001, 0.456)	0.525			
Livers mass				r = 0.372 (0.203, 0.510)	0.062 (+0.015)	4.244 (<0.001)
Combined testes mass				r = 0.214 (0.031, 0.376)	0.024 (+0.010)	2.321 (0.022)
SVL				r = −0.120 (−0.293, 0.066)	−0.065 (+0.051)	−1.274 (0.205)
3. Size asymmetry	116	0.119 (<0.001, 0.421)	0.401			
ASR				r = −0.063 (−0.245, 0.126)	−0.009 (+0.014)	−0.653 (0.515)
SVL				r = 0.622 (0.499, 0.711)	0.208 (+0.025)	8.211 (<0.001)
4. Size asymmetry	116	0.103 (<0.001, 0.348)	0.401			
Mating system				r = −0.124 (−0.296, 0.061)	−0.015 (+0.011)	−1.324 (0.188)
SVL				r = 0.628 (0.511, 0.713)	0.201 (+0.023)	8.581 (<0.001)
5. Size asymmetry	116	0.084 (<0.001, 0.363)	0.505			
Body fat				r = 0.317 (0.141, 0.464)	0.038 (+0.109)	3.531 (<0.001)
Combined testes mass				r = 0.218 (0.035, 0.379)	0.025 (+0.011)	2.361 (0.020)
SVL				r = −0.070 (−0.247, 0.115)	−0.038 (+0.051)	−0.739 (0.462)
6. Size asymmetry	116	0.119 (0.002, 0.409)	0.466			
Lung mass				r =0.179 (−0.005, 0.346)	0.020 (+0.011)	1.925 (0.057)
Combined testes mass				r = 0.271 (0.092, 0.425)	0.033 (+0.011)	2.977 (0.004)
SVL				r = 0.063 (−0.122, 0.241)	0.032 (+0.048)	0.663 (0.509)
7. Size asymmetry	116	0.130 (0.006, 0.407)	0.448			
Latitude				r = −0.022 (−0.203, 0.162)	−0.0002 (+0.001)	−0.229 (0.819)
Combined testes mass				r = 0.316 (0.141, 0.464)	0.039 (+0.011)	3.530 (0.001)
SVL				r = 0.176 (−0.009, 0.343)	0.079 (+0.042)	1.889 (0.061)
8. Size asymmetry	116	0.125 (0.004, 0.400)	0.444			
Precipitation seasonality				r = 0.053 (−0.132, 0.233)	0.0002 (+0.0003)	0.558 (0.578)
Temperature seasonality				r = 0.026 (−0.158, 0.207)	0.0007 (+0.0002)	0.273 (0.785)
Combined testes mass				r = 0.301 (0.123, 0.451)	0.037 (+0.011)	3.324 (0.001)
SVL				r = 0.178 (−0.007, 0.346)	0.080 (+0.042)	1.908 (0.059)

## Data Availability

The data presented in this study are available on request from the corresponding author. The data are not publicly available due to privacy or ethical restrictions.

## Supplementary Materials

The following supporting information can be downloaded at: https://www.mdpi.com/article/10.3390/biology12020151/s1, Table S1: Dataset used for all analyses. Sample size (N), latitude, body size (snout-vent length: SVL) (mm), left and right testis mass (mg), livers mass (mg), precipitation seasonality (coefficient of variation), temperature seasonality (standard deviation ×100), mating system and adult sex ratio used for this study. Mating systems are abbreviated as follows: 1 = monandry and 2 = polyandry. Adult sex ratio (ASR: defined as the number of males to the number of fertilizable females); Table S2: Genbank accession numbers of three nuclear genes (RAG1, RHOD and TYR) and the six mitochondrial genes (CYTB, 12S, 16S, COI, ND2 and ND4) used for phylogeny construction; Figure S1: The relationship between adult sex ratio and residual testes size asymmetry across 116 species of anurans. Residual testes size asymmetry was estimated as residuals from the regression of log_10_-transformed SVL and log_10_-transformed testes size asymmetry; Figure S2: Non-significant difference in residual size asymmetry between monandry and polyandry across 116 species of anurans. Residual testes size asymmetry was estimated as residuals from the regression of log_10_-transformed SVL and log_10_-transformed testes size asymmetry; Figure S3: The relationships between residual size asymmetry and latitude across 116 species of anurans. Residual testes size asymmetry was estimated as residuals from the regression of log_10_-transformed SVL and log_10_-transformed testes size asymmetry; Figure S4: The relationships between residual size asymmetry and temperature seasonality (a) and precipitation seasonality (b) across 116 species of anurans. Residual testes size asymmetry was estimated as residuals from the regression of log_10_-transformed SVL and log_10_-transformed testes size asymmetry.
