# Supplementary material for: Testing the Role of Natural and Sexual Selection on Testes Size Asymmetry in Anurans
Source: Biology (Basel). 2023 Jan 18;12(2):151. doi: 10.3390/biology12020151 (PMC9952133; doi:10.3390/biology12020151)

Table S1. Dataset used for all analyses. Sample size (N), latitude, body size (snout-vent length: SVL) (mm), left and right testis mass (mg), livers mass (mg), precipitation seasonality (coefficient of variation), temperature seasonality (standard deviation ×100), mating system and adult sex ratio used for this study. Mating systems are abbreviated as follows: 1= monandry and 2= polyandry. Adult sex ratio (ASR: defined as the number of males to the number of fertilisable females).

Species	N	Latitude	SVL	Precipitation seasonality	Temperature seasonality	Left testis	Right testis	Livers mass	Mating system	ASR
Amolops chunganensis	4	32°41′	35.64	74.74	776.77	16.8	15.2	145.9	1	0.923
Amolops granulosus	4	32°41′	33.28	74.74	776.77	6.97	6.74	85.4	1	0.8
Amolops kangtingensis	4	30°4′	50.66	98.49	604.64	16.41	15.28	119.4	1	0.75
Amolops lifanensis	4	32°31′	52.8	83.77	718.77	35.9	30.4	497.6	1	1.333
Amolops loloensis	4	29°1′	51.42	89.45	588.56	29.88	25.55	911.7	1	1.071
Amolops mantzorum	4	30°32′	53.8	81.24	671.66	67.8	67.4	250.8	1	0.714
Amolops ricketti	4	26°22′	51.36	66.24	736.7	21.07	19.23	466.1	1	1
Amolops sinensis	4	28°37′	52.55	66.24	736.7	79.9	67.2	428.4	1	0.929
Amolops torrentis	4	18°43′	52.56	73.9	310.59	14.51	14.48	159.2	1	1.182
Amolops yunkaiensis	4	22°16′	46.16	73.07	501.11	23.52	19.29	139.2	1	0.85
Aquixalus palpebralis	4	22°54′	27.11	86.42	420.22	1.101	1.099	16.4	2	0.692
Babina daunchina	4	29°35′	48.32	88.66	692.39	48.9	41.7	210.2	1	1.333
Bombina maxima	4	27°43′	58.1	81.33	505	86.9	79.4	441.6	1	0.82
Brachytarsophrys chuannanensis	4	27°17′	102.16	89.09	541.91	662.16	447.14	2328.1	1	0.72
Brachytarsophrys feae	4	26°22′	99.42	61.98	714.21	574.3	412.9	1441.8	1	0.56
Bufo andrewsi	4	30°32′	74.7	81.24	671.66	131.9	100.5	637.2	1	1.273
Bufo gargarizans	4	30°48′	99.5	80.45	764.04	287.3	230.1	3441.3	1	1.444
Bufo melanostictus	4	25°58′	64.43	62.64	750.5	89.7	74.5	381.7	1	1.2
Bufo minshanicus	4	32°41′	67.5	81.19	666.92	141.5	120.2	1172.5	1	1.091
Bufo raddei	4	40°2′	46.22	105.9	1148.4	145.25	89.2	284.6	1	0.714

Bufo tibetanus	4	29°59′	64.87	94.5	621.84	99.6	83.7	1470.3	1	1.167
Calluella yunnanensis	4	24°32′	31.98	78.95	416.81	3.05	2.93	56.2	2	0.8
Chirixalus doriae	4	21°55′	26.1	73.9	310.59	4.58	4.72	81.9	2	
Chirixalus vittatus	4	18°43′	24.14	75.89	350.14	2.8	3	41	2	
Feirana quadranus	4	32°38′	71.1	80.13	765.58	426.2	310.2	986.9	2	1.81
Fejervarya limnocharis	4	28°37′	37.5	86.1	742.51	26.2	24	137.2	1	1.2
Hyla annectans chuanxiensis	4	30°32′	33.88	81.24	671.66	4.97	4.75	48.4	2	0.889
Hyla annectans jingdongensis	5	22°57′	33.5	82.93	438.87	6.75	6.25	45.6	2	1.25
Hyla annectans wulingensis	4	27°52′	33.9	60.44	773.19	1.64	1.67	16.1	2	
Hyla chinensis	4	22°16′	28.2	73.07	501.11	2.12	2.08	46.7	2	1.12
Hyla sanchiangensis	4	27°52′	33.85	60.44	773.19	2.27	2.43	26.6	2	
Hyla tsinlingensis	4	30°36′	34.26	61.13	765.76	4.83	3.37	78.6	2	
Hylarana guentheri	4	30°22′	58.8	86.1	742.51	94.7	90	368	1	1.167
Hylarana nigrotympanica	4	20°0′	45.12	82.02	340.44	6.34	5.89	236.7	1	0.875
Kaloula pulchra	4	20°0′	54.62	82.02	340.44	25.62	18.24	568.3	1	1.071
Kaloula rugifera	4	31°37′	38.9	99.79	725.39	9.7	9.5	116.4	1	0.5
Kaloula verrucosa	4	25°3′	39.2	83.22	435.76	7.78	7.22	119.6	1	0.6
Leptobrachella bijie	4	27°54′	25.36	73.36	717.81	4.34	3.86	18.4	1	0.714
Leptobrachella feii	4	22°56′	22.56	79.78	699.4	2.11	2.09	27.6	1	0.857
Leptobrachium boringii	4	28°42′	60.2	84.83	434.87	37.2	31.3	325.3	1	1.333
Leptolalax oshanensis	4	22°0′	29.59	80.18	331.07	2.96	2.94	24.3	1	0.813
Leptolalax vencruiseunctatus	4	20°0′	25.8	82.02	340.44	3.01	2.99	23.1	1	0.882
Limnonectes fujianensis	4	30°36′	52.58	61.13	765.76	12.2	11.5	216	1	1.333
Lithobates catesbeinaus	4	30°22′	168.52	86.1	742.51	1568.2	969.4	3929.6	1	1.143
Megophrys daweimontis	4	22°56′	44.19	84.83	434.87	23.29	19.97	227.3	1	1.214
Megophrys jingdongensis	4	24°32′	30.22	78.95	416.81	8.38	7.87	38.2	1	1
Megophrys kuatunensis	4	22°16′	29.2	73.07	501.11	2.27	2.23	48.5	1	0.8
Megophrys major	4	22°54′	49.16	86.42	420.22	87.2	65.44	342.4	1	1.333
Megophrys minor	4	29°34′	42.13	91.61	684.5	8.1	7.5	140	1	1

Megophrys omeimontis	4	29°34′	58.42	94.31	692.33	164.23	101.5	478.2	1	1.286
Megophrys parva	4	22°0′	39.22	80.18	331.07	8.79	8.62	75.5	1	0.857
Megophrys shapingensis	4	29°1′	70.61	89.45	588.56	143.21	102.45	2104.1	1	1.24
Megophrys spinata	4	26°24′	47.64	61.44	726.11	81.8	69.6	215.8	1	0.857
Microhyla berdmorei	4	22°0′	26.28	80.18	331.07	8.87	8.65	30.5	1	0.667
Microhyla butleri	4	19°59′	20.95	61.01	748.78	3.8	3.6	22.3	1	0.5
Microhyla heymonsi	4	26°30′	21.42	62.64	750.5	3.6	3.4	16.8	1	0.5
Microhyla mixtura	4	26°30′	20.8	61.01	748.78	5.36	5.64	7.6	*1*	*0.6*
Microhyla ornata	4	30°49′	24.4	81.72	761.3	8.46	8.34	35.5	1	0.556
Microhyla pulchra	4	25°58′	32.58	62.64	750.5	2.33	2.14	43.3	1	0.833
Micryletta inornata	4	21°55′	25.92	75.89	350.14	1.88	2.03	11.4	1	0.9
Nanorana parkeri	4	30°31′	50.27	120.6	708.85	32.29	31.24	86.7	1	0.85
Nanorana pleskei	4	32°58′	31.03	79.64	706.83	30	27.5	121.4	1	0.714
Nanorana ventripunctata	4	27°33′	39.25	62.54	545.58	9.56	8.69	66.3	1	0.444
Nidirana leishanensis	4	26°22′	54.7	61.64	725.31	18.5	16.4	243.1	1	1.182
Nidirana yeae	4	28°36′	54.03	66.12	733.31	24.9	24	139.9	1	1.2
Occidozyga lima	4	19°59′	23.36	80.18	331.07	2.13	2.07	13.1	1	0.8
Occidozyga martensii	4	22°0′	19.8	68.63	410.31	4.75	4.55	22	1	1.125
Odorrana grahami	4	25°3′	67.6	83.22	435.76	29.2	24.3	433	1	1.267
Odorrana graminea	4	28°36′	47.4	73.9	310.59	9.1	9	110.5	1	1.5
Odorrana hainanensis	4	18°43′	52.7	73.9	310.59	10.4	10.3	177.2	1	1.125
Odorrana hejiangensis	4	28°36′	46.6	66	732.34	42.9	36.3	151.3	1	1.304
Odorrana huanggangensis	4	26°22′	44.62	61.85	705.52	45.26	44.37	85.3	1	1.091
Odorrana kuangwuensis	4	32°41′	56.35	74.74	776.77	12.56	12.54	199.4	1	1
Odorrana kweichowensis	4	28°7′	45.12	65.21	749.76	9.83	8.76	151.6	1	1.182
Odorrana lungshengensis	4	26°22′	65.29	61.98	714.21	78.92	60.45	586.2	1	1.222
Odorrana margaretae	4	28°7′	72	65.21	749.76	28.7	27.8	643.9	1	1.263
Odorrana nanjiangensis	4	32°35′	57.53	82.66	777.95	22.5	19.7	364.7	1	1.4
Odorrana nasuta	4	29°12′	59.45	91.78	691.19	16.3	15.9	230.9	1	1.25

Odorrana schmackeri	4	22°54′	47.7	86.42	420.22	25.6	22.3	279.5	1	1.2
Odorrana tiannanensis	4	18°43′	61.25	73.9	310.59	27.16	22.35	572.3	1	1.636
Ophryophryne microstoma	4	22°57′	30.26	81.21	444.96	3.42	3.21	45.5	1	1.167
Oreolalax rugosus	4	28°55′	44.92	92.28	548.69	11.76	9.18	141.9	1	0.64
Paa boulengeri	4	29°36′	73.5	93.53	695.33	203.6	144.5	803.9	1	1.286
Paa robertingeri	4	28°36′	77.48	66	732.34	155.4	126.1	686.2	1	1.077
Paa sichusnensis	4	25°57′	85.14	65.65	449.76	281.9	176.2	1913.9	1	1.25
Paa yunnanensis	4	25°3′	60.6	83.22	435.76	258.6	192.1	696.2	1	0.938
Pelophylax fukienensis	4	26°7′	51.1	59.54	667.41	24.9	22.5	211.5	1	0.68
Pelophylax hubeiensis	4	29°47′	41.8	70.99	743.08	90.5	77.2	280.2	1	1.167
Pelophylax nigromaculatus	4	30°50′	67.9	81.48	759.14	149.6	141.1	566.2	1	1.667
Pelophylax pleuraden	4	25°3′	54.5	83.22	435.76	16.4	14.4	347.9	1	1.241
Philautus gracilipes	4	23°0′	28.72	81.39	442.45	4.25	4.13	19.7	1	0.538
Philautus jinxiuens	4	22°54′	27.5	86.42	420.22	6.4	5.7	61	1	0.75
Philautus odontotarsus	4	23°	30.02	81.39	442.45	7.01	6.99	22.1	2	
Polypedates braueri	4	23°1′	45.73	79.28	446.59	30.5	28.2	98.9	2	1.091
Polypedates impresus	4	22°45′	45.38	87.75	364.93	55.2	53.9	102.4	2	1.167
Polypedates megacephalus	4	29°50′	43.6	69.25	742.61	51.7	48.1	62.3	2	1
Polypedates mutus	4	25°58′	40.1	62.64	750.5	61.8	58.4	64.7	2	1.4
Pseudorana weiningensis	4	26°22′	36.22	61.85	705.52	17.3	15.9	47.8	1	0.583
Rana chaochiaoensis	4	27°10′	50.4	97.1	497.94	12.7	12.6	85.4	1	1.375
Rana chensinensis	4	29°8′	44.3	61.69	744.89	15.9	14.2	122.7	1	1.333
Rana hanluica	6	26°22′	48.41	61.64	725.31	5.58	4.35	137.2	1	0.875
Rana kukunoris	4	32°34′	49.4	80.45	674.69	75.1	61.3	272.4	1	1.182
Rana omeimontis	6	29°36′	51.48	93.53	695.33	15.9	14.68	141.6	1	1.185
Rhacophorus chenfui	4	29°36′	38.6	93.53	695.33	62.2	54.3	164.3	2	1.167
Rhacophorus dennysi	4	27°52′	74.89	60.44	773.19	235.2	192.4	338.4	2	1.588
Rhacophorus dugritei	4	29°35′	42.2	88.66	692.39	27.1	25.3	100.9	2	1.5
Rhacophorus feae	4	22°56′	104.35	84.83	434.87	1017.1	775.1	1016	2	0.97

Rhacophorus hongchibaensis	4	33°6′	65.78	76.46	832.45	51.26	51.04	88.6	2	1.108
Rhacophorus hungfuensis	4	30°55′	35.12	89.43	691.68	32.5	31.1	121.1	2	1.72
Rhacophorus omeimontis	4	30°32′	59.8	81.24	671.66	187.1	180.8	192.3	2	0.98
Rhacophorus rhodopus	4	20°0′	37.51	82.02	340.44	24.5	23.4	128.2	2	0.82
Scutiger chintingensis	4	29°31′	44.28	80.04	632.15	85.2	75.8	158.2	1	0.6
Scutiger glandulatus	4	31°43′	66.11	78.24	645.01	67.9	54	1312.7	1	0.9
Scutiger muliensis	4	28°33′	72.18	91.41	564.57	119.2	85.8	2978.3	1	1.2
Xenophrys leishanensis	4	26°22′	35.55	61.98	714.21	17.2	16.3	67.4	1	1
Xenophrys wushanensis	4	30°36′	52.16	61.13	765.76	68.1	63.6	233.5	1	1.4

Table S2. Genbank accession numbers of three nuclear genes (RAG1, RHOD and TYR) and the six mitochondrial genes (CYTB, 12S, 16S, COI, ND2 and ND4) used for phylogeny construction.

Species	12S	16S	COI	CYTB	ND2	ND4	RAG1	RHOD	TYR
Amolops chunganensis	KX645666	KX645666	KX645666	KX645666	KX645666	KX645666	KU840719		KU840752
Amolops granulosus	KF771286	MK573802	NC 044901	KJ008444	NC 044901	KF771306			
Amolops kangtingensis	EF453727	EF453742	JN700800	KJ008423	KF771330	KF771307	EF088240	DQ360023	EU076762
Amolops lifanensis	DQ359981	MK573810	MK605628	KJ008458	KX269407			DQ360034	DQ360065
Amolops loloensis	KF771288	KT750963	KT750963	KT750963	KY357479	KT750963	KX208690		KY357486
Amolops mantzorum	DQ359970	MK604858	KJ546429	KJ008405	KJ546429	KF771309	EF088240	DQ360023	DQ360054
Amolops ricketti	NC_023949	KF956111	KF956111	KF956111	KF956111	KF771310	KX208724	DQ360009	EU076764
Amolops sinensis		MK263279	MK263320		MN958804				
Amolops torrentis	MG991872	DQ204489	MG991932		MN958785		KU840715		
Amolops yunkaiensis	MG991847	MK263276	MG991913						
Aquixalus palpebralis	EU924625	GQ285681	DQ468683	EU924597			GQ285773	GQ285792	GQ285810
Babina daunchina	KU840524	MF807825	HQ395353	KF020631			KU840723		KU840782
Bombina maxima	DQ925758	JX893181	JX893181	EU531274	JX893181	EU789363			
Brachytarsophrys chuannanensis	MH406636	KM504261	MT162631	MH407195			MH405148		
Brachytarsophrys feae	KY022194	KY022194	MH406362	MH407193			AY523740	KX812393	
Bufo andrewsi	AF160764	FJ882808	MF805611	AF174502	AY607301		DQ158353	DQ283905	
Bufo gargarizans	AF205541	FJ882843	KY385685	AF171196	KU321581	DQ275350	KF666177		

Bufo melanostictus	AB167899	KJ697684	AJ584640	AF249082	KT031590	DQ904392	KT031693	AF249097	
Bufo minshanicus	KM587710	KM587710	KY385799	KM587710	KM587710	KM587710			
Bufo raddei	KF664854	KR861720	KR861720	AF171210	KY555701	KR861720	KF666186		
Bufo tibetanus	AF160766	JX878885	HM582424	AF171193	KX269407	JX878885			
Calluella yunnanensis	AB634626	KM509102	KM509769						KM509902
Chirixalus doriae	EF564446	AB813159	KR087631	GQ204538			GQ204602	GQ204657	DQ347165
Chirixalus vittatus	EF564448	DQ283134	KR087649	EU924596			GQ204603	GQ204658	EU924568
Feirana quadranus	GQ225906	AB200967			GQ225933		HM163591	EU979886	EU979981
Fejervarya multistriata	AB277282	KR071859	KR071859	AB296096	KR071859	KR071859	AB526660	DQ458271	AB500265
Hyla annectans chuanxiensis	KP742565	KP742695		FJ226919					
Hyla annectans jingdongensis	KP742564	KP742693	JN700883	AY843821			AY844388	AY844574	AY844045
Hyla annectans wulingensis	KP742563	KP742692							
Hyla chinensis	AY458593	AY458593	AY458593	AY458593	AY458593	AY458593	KX208743		
Hyla sanchiangensis	MT561180	MT561180	MT561180	MT561180	MT561180	MT561180			
Hyla tsinlingensis	KP742646	KP212702	KP212702	JX870448	KP212702	KP212702			
Hylarana guentheri	KF185024	KR264039	KM035413	KM035413	KM035413	KM035413	KX208782	AY322216	KR264440
Hylarana nigrotympanica		DQ650392	KR087688		FJ417171		EF088267		EU076793.1
Kaloula pulchra	AY458595	DQ283398	AY458595	AY458595	AY458595	AY458595	KX208748	DQ284011	KC180289
Kaloula rugifera	NC_029409	KT878719	KT878719	KT878719	KT878719				
Kaloula verrucosa	JX678893	NC 039411	NC 039411	NC_039411	NC 039411	NC 039411			
Leptobrachella bijie		MK414535							
Leptobrachium boringii	KJ630505	MH643882	KJ630505	MH643882	KJ630505	KJ630505	MH056123	KX812400	
Leptobrachella feii		MT302635							
Leptolalax oshanensis	NC_020610	KX811928	KC460337	KC460337	KC460337	KC460337	MH056094	MH056153	
Leptolalax ventripunctatus	LC201979	KX811929	KR087772				MH056090	MH056133	
Limnonectes fujianensis	AY974191	HM067232	NC 007440	FJ349544	NC 007440	NC 007440	KX208751	DQ458260	DQ458275
Lithobates catesbeianus	MN241124	DQ289127	AB761267	AF205089	AF314016	MN241124	AB612037	S79840	DQ360044
Megophrys daweimontis		KX811868	KX812124				KX812250	KX812362	
Megophrys jingdongensis	MH406617	KX811875	KR087946	MH407176			MH405129	KX812354	
Megophrys kuatunensis	MF667850	MN563765	MF667916	MH407036			MH404982		
Megophrys major	KY022311	DQ283374	KR087949	MH407011			MH405013		DQ282986
Megophrys minor	MH406474	KJ560389	MF667893	MH407028			MH404973	KX812325	
Megophrys omeimontis	EF397242	EF397242	KP728257	AY561314	KX021971	KP728257	EF397280	KX812347	

Megophrys parva	MH406568	KY022217	MH406297	MH407127			MH405078		
Megophrys shapingensis	NC_018785	JX458090	JX458090	AY561310	MT561180	JX458090	KX812274	KX812317	
Megophrys spinata		KX811864	KX812120	AY561317			KX812231	KX812349	
Microhyla berdmorei	AB634603	LC465690	KR087785	LC465615			LC465764	KU840621	KU840794
Microhyla butleri	KT285802	KT285802	KT972718	KT972718	KT972718	KT285802	EF396094		
Microhyla heymonsi	AY458596	MT808958	AY458596	AY458596	AY458596	AY458596	KX208756		EF395979
Microhyla mixtura	NC_038130	LC465669	NC 038130	NC_038130	NC_038130	NC 038130	LC465744		
Microhyla ornata	JN541317	DQ512876	DQ512876	AB201223	DQ512876	DQ512876	AY364198	AY364383	KC180221
Microhyla pulchra	NC_024547	LC465692	KF798195	KF798195	KF798195		EF396093	KU840623	EF395980
Micryletta inornata	KC822493	KM509167	AB611965				EF396096		EF395981
Nanorana parkeri	EU979723	KP317482	KP317482	KJ434188	GQ225878	KP317482	HM163584	EU979872	DQ458276
Nanorana pleskei	EU979781	HQ324232	HQ324232	HQ324232	GQ225879	HQ324232	HM163586	EU979871	EU979962
Nanorana ventripunctata	EU979717	NC 039094	NC 039094	NC_039094	NC_039094	NC 039094	HM163585	EU979866	EU979959
Nidirana leishanensis	MK293797	MN946454	MN945210						MK293864
Nidirana yeae		MN295230	MN295238						
Occidozyga martensii	GU177877	DQ283357	GU177877	GU177877	GU177877	GU177877	KU243099	DQ283978	KU243119
Occidozyga lima	AB488880	DQ283224	KR087832	AB488857			AB488961	AB489042	AB489018
Odorrana grahami	KU840529	KF185051	MH697567		EU861580	KF771313	EF088257	DQ360016	DQ360047
Odorrana graminea	NC_050884	KF185037	NC 050884	NC_050884	KR338281	NC 050884	KP221672	KU840632	KP191588
Odorrana hainanensis	NC_034984	KX056491	KX056491	KX056491	KX056491	KX056491	KU840725	KU840664	
Odorrana hejiangensis	KU840531	KF185052			KR338211		KU840727	KU840683	KU840788
Odorrana huanggangensis	MK650099	KT315387			MH193613				
Odorrana kuangwuensis	KF184998	KF185034		KU217313					
Odorrana kweichowensis	MH193535	MH193548			MH193602				
Odorrana lungshengensis	KF185018	KF185054	KF771336		KX269407				
Odorrana margaretae	DQ359964	KF185035	KJ815050	KU217223	EU861592	KJ815050	EF088261	DQ360017	DQ360048
Odorrana nanjiangensis	KF185006	KF185042							
Odorrana nasuta	KF185017	KF185053							
Odorrana schmackeri	KP732086	KF185047	KJ149452	KP732086	GU812150	KP732086	KX208784	DQ360020	KP191589
Odorrana tiannanensis	KF185008	KF185044	KR087846						
Ophryophryne microstoma	MH406565	DQ283391	MK524157	MH407134			KY022234	KX812398	
Oreolalax rugosus	EF397254	EF397254							
Paa boulengeri	EU979791	KC686711	KX233867	JX676597	KX233867	KF199152	HM163604	EU979918	EU980033

Paa robertingeri	KY441640	KY441640	KY441640	KY441640	GQ225880	KY441640		DQ458265	DQ458280
Paa sichuanensis	KU139984	Odorrana kweichowensis							
Paa yunnanensis	GQ225869	KF199150	KF199150	KF199150	GQ225878	KF199150	HM163593	DQ458263	EU979976
Pelophylax fukienensis	AB029941		MH034303	JQ995141					GU978236
Pelophylax hubeiensis	AF205547	AF315137		JQ995009					
Pelophylax nigromaculatus	AB705490	KX269216	KT878718	DQ006266	GU812150	KT878718	AB360184	DQ283838	DQ360045
Pelophylax pleuraden	JN541324	JQ621943	HQ395289	KR264150		KF771303	KR264384	DQ360011	DQ360042
Philautus gracilipes	EF564451	DQ283051	KR087672	EU924593			GQ285764	DQ283780	GQ285807
Philautus jinxiuensis	EF564452	EU215525	KR087673	EU924599			GQ285763	EU924543	EU924571
Philautus odontotarsus	EF564460	AB933303	KX554578	EU924594	DQ287141		KX208772	EU215579	KX554685
Polypedates braueri	NC_042797	AB728007	KT921226	NC_042797	NC 042797	NC 042797	AB728251	AB728274	AB728308
Polypedates impresus	MK481034	KF053209	KR087863					MW266002	MW269977
Polypedates megacephalus	KU840483	KC465836	AY458598	AB451722	NC 043955	NC 043955	EU924517	EU924545	KC180271
Polypedates mutus	AY880608	MW266017	MG935684	MK622900		MK622900	GQ285770	EU215581	EU215611
Pseudorana weiningensis	DQ359959	KX269217		KX269362	KX269432		KX269582	DQ360012	DQ360050
Rana chaochiaoensis	DQ359975	KX269192	KU246048	AF274927	KU246048	KF771302	KX269557	DQ360028	DQ360059
Rana chensinensis	AB058853	KF185061	MT418625	AF077396	GU812150	KF771317	KX269551	DQ360030	DQ360061
Rana hanluica	KX269191	KX269191	MT418638	KX269338	KX269407		KX269556		KX269784
Rana kukunoris	KX269185	KU246049	MN733918	JX486345	MN733918	MN733918	GQ285780	GQ285798	GQ285816
Rana omeimontis	DQ289083	KU246050	KU246050	AF274928	KU246050	KU246050	KX269558		KX269785
Rhacophorus chenfui	GQ204763	JX219431	KP996734	EU924603			EU924519	EU924547	KU840751
Rhacophorus dennysi	DQ019592	KT191129	LC386527	EU924604	KT191129	KX233869	DQ019512	EU215575	EU924576
Rhacophorus dugritei	EF564471	LC010584	KP996709	EU924605	MK357046		GQ285768	EU215571	EU215601
Rhacophorus feae	EF564474	EU215544	LC386530	EU924606			EU924522	EU924550	EU924578
Rhacophorus hongchibaensi	JN688882	JN688882						JN688898	JN688907
Rhacophorus hungfuensis	EU215538	EU215538	KP996733		MK357046			JN688903	JN688913
Rhacophorus omeimontis	EF564493	NC 046387	NC 046387	EU924612	KP895687	NC 046387	EU924528	EU215565	KU840753
Rhacophorus rhodopus	EF564507	JX219439	LC386538	EU924616			EU924532	EU215561	EU924588
Scutiger chintingensis	EF397270	EF397270	KY310878	KY310930		KY310991	KY311043		
Scutiger glandulatus	EF397276	EF397271	KY310883	KY310935		KY310995	KY311049		
Scutiger muliensis	EF397277	EF397272					EF397302		
Xenophrys leishanensis		MK005310					MK005318		
Xenophrys wushanensis	MH406469	MH406736	MH406188	MH407018			MH404963	KX812319	

Figure S1. The relationship between adult sex ratio and residual testes size asymmetry across 116 species of anurans. Residual testes size asymmetry was estimated as residuals from the regression of log_{10}-transformed SVL and log_{10}-transformed testes size asymmetry.

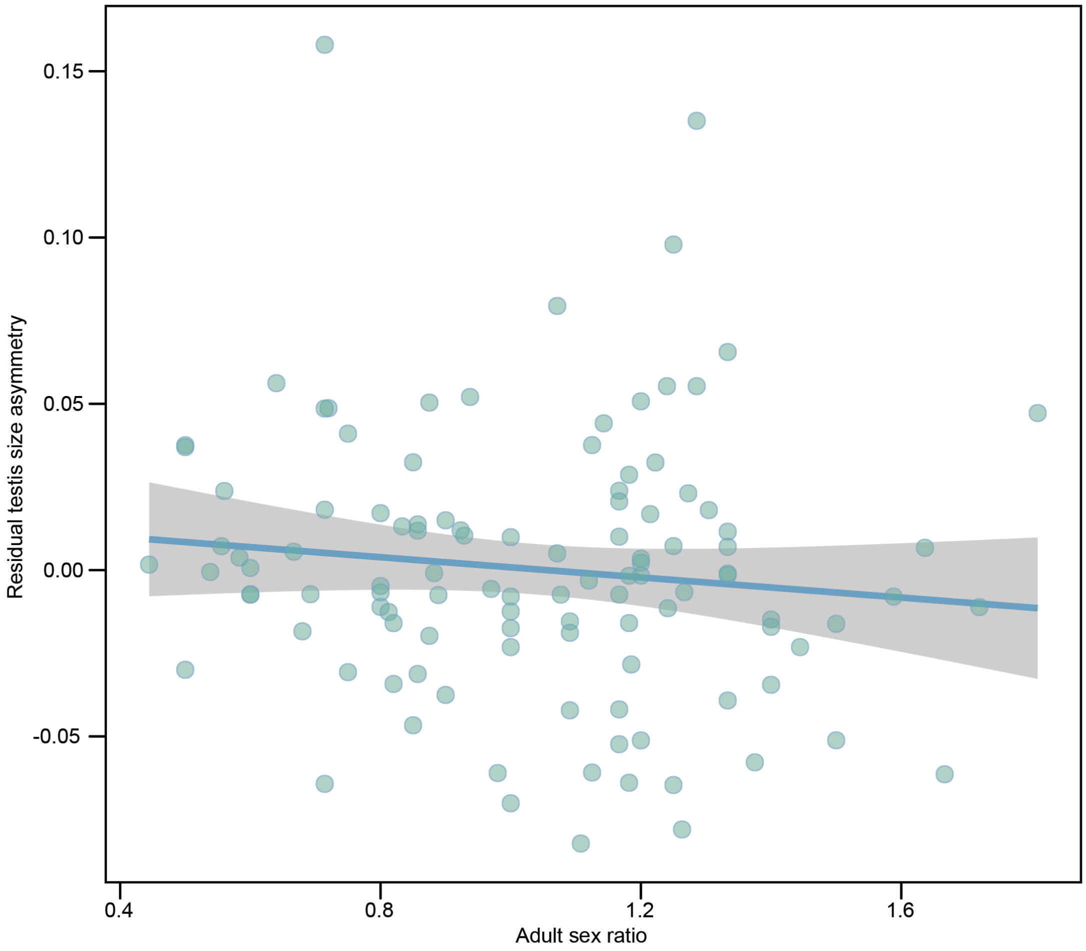

Figure S2. Non-significant difference in residual size asymmetry between monandry and polyandry across 116 species of anurans. Residual testes size asymmetry was estimated as residuals from the regression of $\log_{10}$-transformed SVL and log-transformed testes size asymmetry.

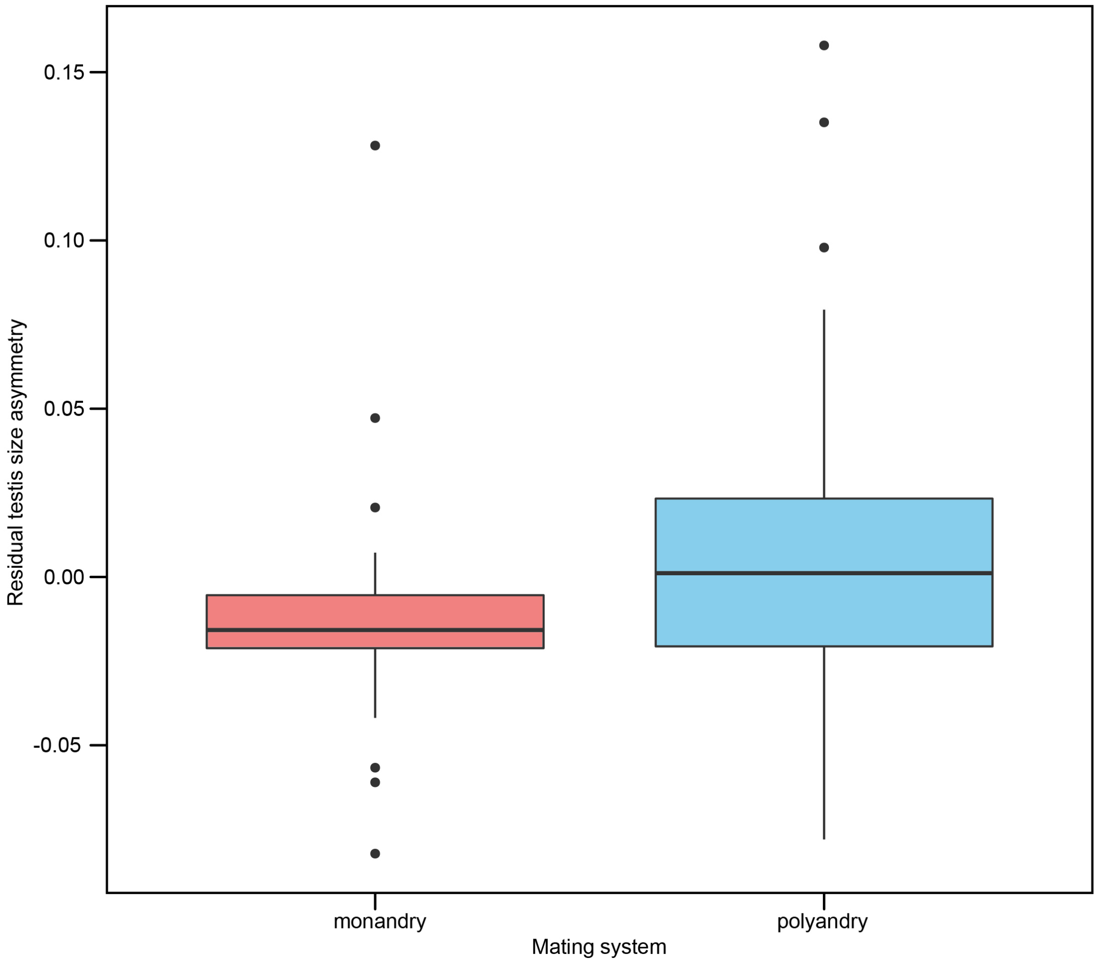

Figure S3. The relationships between residual size asymmetry and latitude across 116 species of anurans. Residual testes size asymmetry was estimated as residuals from the regression of log_{10}-transformed SVL and log_{10}-transformed testes size asymmetry.

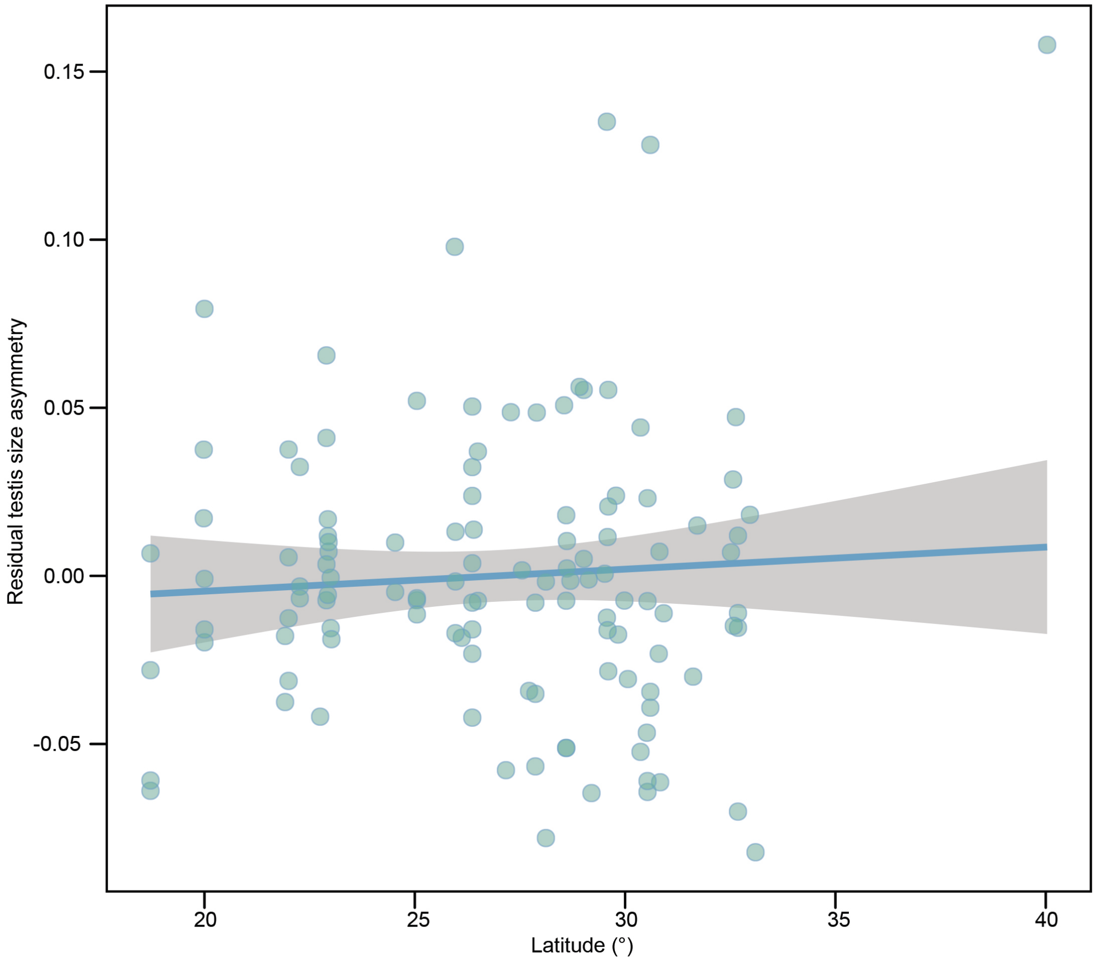

Figure S4. The relationships between residual size asymmetry and temperature seasonality (a) and precipitation seasonality (b) across 116 species of anurans. Residual testes size asymmetry was estimated as residuals from the regression of $\log_{10}$-transformed SVL and $\log_{10}$-transformed testes size asymmetry.

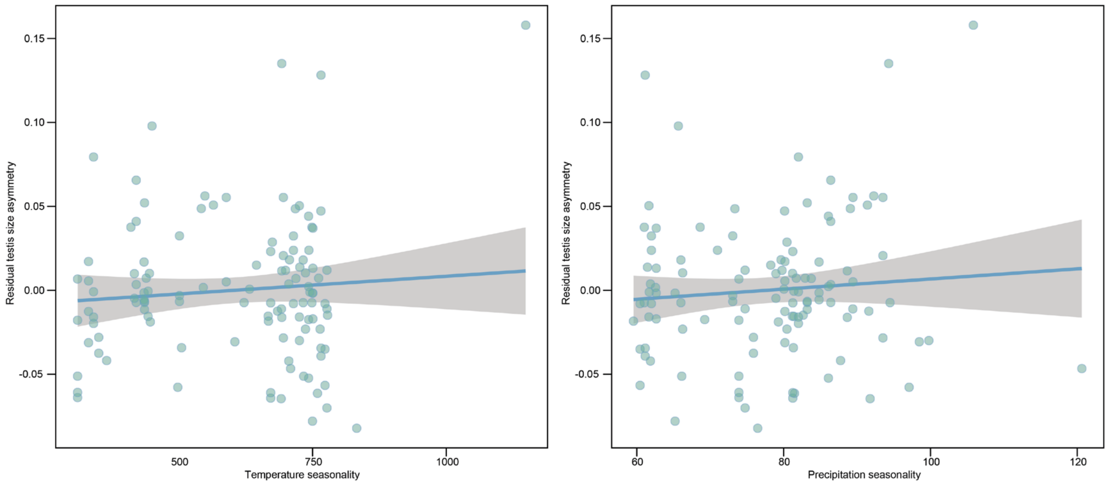

Supplement: Supplementary file 1 [file biology-12-00151-s001.zip › biology-2103577-supplementary.pdf]
